# Inequalities in access to safe drinking water in Peruvian households according to city size: an analysis from 2008 to 2018

**DOI:** 10.1186/s12939-021-01466-7

**Published:** 2021-06-05

**Authors:** Akram Hernández-Vasquéz, Carlos Rojas-Roque, Denise Marques Sales, Marilina Santero, Guido Bendezu-Quispe, Tonatiuh Barrientos-Gutiérrez, J. Jaime Miranda

**Affiliations:** 1 CRONICAS Center of Excellence in Chronic Diseases, Universidad Peruana Cayetano Heredia, Av. Armendariz 497, Miraflores, 12 Lima, Peru; 2 Universidad de Buenos Aires, Buenos Aires, Argentina; 3 Observatory for Urban Health in Belo Horizonte, Federal University of Minas Gerais, Belo Horizonte, Brazil; 4 School of Public Health and Administration, Universidad Peruana Cayetano Heredia, Lima, Peru; 5 National Institute of Public Health, Center for Population Health Research, Cuernavaca, Mexico

**Keywords:** Socioeconomic factors, Inequality, Drinking water, Cities, Latin America, Peru

## Abstract

**Background:**

Peru is one of the countries with the lowest percentage of population with access to safe drinking water in the Latin American region. This study aimed to describe and estimate, according to city size, socioeconomic inequalities in access to safe drinking water in Peruvian households from 2008 to 2018.

**Methods:**

Secondary analysis of cross-sectional data using data from the 2008–2018 ENAHO survey. Access to safe drinking water, determined based on the presence of chlorinated water supplied by the public network, as well as socioeconomic variables were analyzed. A trend analysis from 2008 to 2018, and comparisons between 2008 versus 2018 were performed to understand and describe changes in access to safe drinking water, according to city size. Concentration curves and Erreygers concentration index (ECI) were estimated to measure inequalities in access to safe drinking water.

**Results:**

In 2008, 47% of Peruvian households had access to safe drinking water, increasing to 52% by 2018 (*p* for trend < 0.001). For small cities, access to safe drinking water did not show changes between 2018 and 2008 (difference in proportions − 0.2 percentage points, *p* = 0.741); however, there was an increase in access to safe drinking water in medium (difference in proportions 3.3 percentage points, *p* < 0.001) and large cities (difference in proportions 12.8 percentage points, *p* < 0.001). The poorest households showed a decreasing trend in access to safe drinking water, while the wealthiest households showed an increasing trend. In small cities, socioeconomic inequalities showed an increase between 2008 and 2018 (ECI 0.045 and 0.140, *p* < 0.001), while in larger cities, socioeconomic inequality reduced in the same period (ECI: 0.087 and 0.018, *p* = 0.036).

**Conclusions:**

We report a widening gap in the access to safe drinking water between the wealthiest and the poorest households over the study period. Progress in access to safe drinking water has not been equally distributed throughout the Peruvian population. Promoting and supporting effective implementation of policies and strategies to safe drinking water, including equity-oriented infrastructure development and resource allocation for most vulnerable settings, including emerging small cities, is a priority.

**Supplementary Information:**

The online version contains supplementary material available at 10.1186/s12939-021-01466-7.

## Background

In 2010, the United Nations General Assembly explicitly recognized access to clean drinking water and sanitation as a human right [1]. However, as of 2015, 3 out of 10 people worldwide did not have access to safe, readily available water at home [2]. Inadequate water, sanitation and hygiene, a major global health problem, accounts for a large part of diarrheal diseases, malaria, schistosomiasis, anemia, malnutrition and death in developing countries [3]. In addition to the health-related burden, there are additional costs linked to collecting water, impaired productivity and economic losses, among others [2, 4, 5]. In response to this phenomena, achieving universal and equitable access to safe drinking water for all is reflected in the Sustainable Development Goal (SDG) 6 on water and sanitation [6].

In 2015, 95% of the Latin American and the Caribbean (LAC) population used an improved drinking water source. Access to drinking water has increased between 1990 and 2015 in both urban and rural regions of LAC, yet 34 million people still lack access [7]. However, the coverage in the access to drinking water in the rural areas, in general, is considerably lower compared to urban areas of LAC [7]. Inequalities in the access to drinking water and public services is also described in smaller cities compared to large urban centers and within urban areas to the detriment of the poorest. Likewise, large gaps in access to improved sanitation and drinking water exist between rich and poor and between geographic regions and across countries [7]. A multi-country study found income inequalities in access to and the use of drinking water services in LAC, and also found that access to household water disinfection methods is very limited among poor families due to its relatively high cost, which results in poorer quality of drinking water in the lower-income groups [8].

Peru ranks third among LAC countries with the most renewable freshwater resources [9]. However, this abundant supply does not reach everyone. The percentage of the population with access to chlorinated water in Peru is 87%, a lower value in comparison to the regional average of 95% for South America [10]. National estimates from Peru’s National Household Survey (ENAHO, by its acronym in Spanish) for 2016 reported that 86% had access to water through the public network, 67% as drinking water and 19% as non-drinking water, and 14% reported consumption of non-potable water from other sources such as rivers, springs, rain, tanker or “pilon” for public use [11]. Access to drinking water according to place of residence also reveals drastic marked differences: 85% in urban areas vs. 9% in rural areas [11]. Furthermore, it is reported that only 77.5% of the water distributed to small cities in Peru is chlorinated [12].

In Peru, both small and large cities have expanded in recent decades, and most of this expansion was unplanned. In addition, the residential segregation by class and ethnicity may produce inequalities within cities, affecting the health of their citizens [12]. Therefore, identifying and measuring inequalities within cities represent an opportunity to improve population health. Unfortunately, there is limited evidence measuring inequalities by different population subgroups or across different geographical regions according to the city size to guide adequate policies.

The aim of this study is to describe and estimate, according to city size, socioeconomic inequalities in access to safe drinking water in Peruvian households from 2008 to 2018.

## Methods

### Study design

A secondary analysis of cross-sectional data from the 2008–2018 ENAHO was carried out. These surveys have been executed by the Peruvian National Institute of Statistics and Informatics (INEI, by its acronym in Spanish). The ENAHO datasets per year used in this study are open access and can be obtained at the INEI website: http://iinei.inei.gob.pe/microdatos.

### Study participants and survey characteristics

We used data from ENAHO, an annual cross-sectional household survey that collects information about the living conditions of the Peruvian population using a multistage, stratified, probabilistic sampling of areas with representation at the national, regional administrative subdivisions (24 regions), natural region (Coast, Highlands, and Jungle), geographic domain (Coast Urban, Coast Rural, Highland Urban, Highland Rural, Jungle Urban, Jungle Rural, and Lima Metropolitan), and urban/rural levels. Sampling is defined as the set of all the private homes and their resident occupants. Only data between 2008 and 2018 were analyzed. Sample sizes ranged from 26,010 in 2008 to 47,700 households in 2018. To produce population-based estimates, the household records were assigned a sampling weight. Weights were designed to minimize bias by incorporating adjustments for various forms of survey nonresponse. Further details on sampling design, data collection, and data quality have been described and published in ENAHO reports [13].

### Outcome

The study’s outcome was access to safe drinking water (yes/no), determined based on the presence of chlorinated water supplied by the public network. This outcome was measured in the general population and according to city size [13]. Regarding city size, based on the classification used by the Peru’s INEI, three categories were defined according to the number of households: small cities (from 401 to 10,000 households), medium cities (from 10,001 households to 100,000 households), and large cities (from 100,001 households or more). Areas with 400 or less households are considered as rural by INEI, and these were not considered since the study was focused in urban cities.

Regarding the measure of water chlorination levels, the surveyor filled two 5 mL tubes with the water used by the household member to wash or prepare their food. The first tube acted as a control. One DPD1 (diethyl paraphenylene diamine) pellet was added to the second tube, and then it was shaken. The two tubes were put on the Free chlorine color disc test kit, model CN-66F equipment to read the results. Water was classified as safe drinking water when the level of free chlorine indicated by the notch in the window of the scale of the test kit was equal or more than 0.5 mg/L.

### Exposure

The exposure variable was the household’s living standards. The exposure variable was derived from per capita household expenditure, measured in Peruvian Soles (PEN), and obtained from the total household expenditure divided by the number of household members. Then, we created quintiles according to the per capita household, where the lowest quintile (Q1 and Q2) are the poorest households and the highest quintile (Q4 and Q5) are the richest households.

### Covariates

For this study, covariates such as natural region (Lima Metropolitan, Rest of the Coast [Coast natural region excluding Lima Metropolitan], Highlands, Jungle), household size (1–3 members, 4–6 members, > 6 members) and poverty at the household level (extreme poor, non-extreme poor, non-poor) were considered. The level of household poverty was classified according to the INEI methodology that considers the household consumption patterns of 941 products. Further details can be consulted in ENAHO reports [13].

### Data analysis

A descriptive analysis was carried out to characterize the Peruvian households surveyed according to socioeconomic characteristics and city size. In addition, the comparison of proportions in access to safe drinking water between 2008 versus 2018 was made using the *lincom* command in Stata. Also, to describe trends in access to safe drinking water, we performed a trend analysis for proportions using Royston’s “*ptrend*” Stata module, [14] and additional analyses using logistic regressions were conducted to re-confirm our results.

Measurements of inequalities in access to safe drinking water were performed using concentration curves (CC) and Erreygers concentration index (ECI) [15]. Both measures are widely used to estimate absolute inequalities using population surveys, as well as to compare differences across years [16]. CCs describe the relationship between the cumulative percentage of the population according to per capita household expenditure and the cumulative percentage of access to safe drinking water in relation to the diagonal line of equality. The more the CC moves away from the equality line, the greater degree of inequality. If the CC is below the equality line, there is greater access to safe drinking water for the richest households (if CC is upper the equity line indicates greater access in the poorest household). ECI was used to measure the magnitude of inequality of access to safe drinking water. This index offers methodological advantages compared to the standard inequality index [15]. The ECIs values range between − 1 and 1, having positive values when there is greater access to water for richest households (negative ECI values indicate greater access in the poorest households).

Statistical analysis were carried out using Stata v14.2 (Stata Corporation, College Station, Texas, USA) specifying the characteristics of the survey sampling that include the weights according to strata study design (*svy* command). All estimates were calculated together with their 95% confidence intervals (95% CI).

### Ethics statement

Approval by an ethics committee was not required to conduct this study because it is a secondary analysis of data obtained from a publicly available source (ENAHO surveys), which does not include personal identifiers of the people surveyed.

## Results

The analysis included 400,677 households from a period of 11 years of ENAHO across the country. Table 1 shows the distribution of Peruvian households according to their socioeconomic characteristics and city size. From 2008 to 2018, the proportion of households with one to three members increased from 33 to 40%, while the proportion of households with more than six members remained constant. In the same period, the median household per capita expenditure increased from PEN 4271 to PEN 7061, whereas a reduction was observed in the proportion of households classified as extremely poor (from 9 to 2%) and non-extreme poor (from 22 to 14%).

**Table 1:** Distribution of Peruvian households according to their socioeconomic characteristics and city size, ENAHO 2008–2018

	Overall (excluding rural areas)	Natural region				City size (number of households)			Household size (members)			Household per capita expenditure, measured in Peruvian soles (S/)	Poverty level in the household		
Year		Lima Metropolitan	Rest of the Coast	Highlands	Jungle	401 to 10,000	10,001 to 100,000	≥ 101,001	1–3	4–6	> 6	Median (Q1-Q3)	Extreme poor	Non extreme poor	Non poor
2008															
n (%)	26,010 (100.0)	3279 (31.4)	6579 (22.9)	10,724 (34.1)	5428 (11.6)	14,952 (42.8)	6737 (20.1)	4321 (37.0)	8759 (33.4)	9990 (39.0)	7261 (27.6)	4270.6 (2451.6–6915.8)	2594 (8.8)	5128 (22.4)	13,780 (68.7)
2009															
n (%)	26,598 (100.0)	3333 (32.2)	6616 (22.3)	11,116 (34.1)	5533 (11.4)	15,399 (42.4)	6843 (19.8)	4356 (37.8)	8852 (33.0)	10,278 (38.7)	7468 (28.2)	4443.1 (2574.9–7349.7)	2396 (8.0)	4926 (20.3)	14,431 (71.7)
2010															
n (%)	27,176 (100.0)	3353 (32.4)	6684 (21.8)	11,424 (34.4)	5715 (11.4)	15,814 (41.9)	6961 (19.9)	4401 (38.1)	9000 (32.9)	9997 (36.9)	8179 (30.2)	4815.3 (2851.0–7700.4)	1804 (6.2)	4583 (19.4)	15,109 (74.3)
2011															
n (%)	32,519 (100.0)	3796 (31.2)	8510 (22.0)	13,510 (34.8)	6852 (11.9)	19,137 (43.4)	8277 (19.5)	5105 (37.1)	10,767 (32.8)	11,322 (35.4)	10,430 (31.9)	5182.8 (3121.7–8114.3)	1751 (5.2)	4841 (17.9)	18,217 (76.9)
2012															
n (%)	32,546 (100.0)	3815 (31.5)	8467 (21.3)	13,425 (35.0)	6839 (12.1)	19,034 (43.3)	8422 (19.7)	5090 (37.0)	11,235 (33.9)	11,302 (34.4)	10,009 (31.7)	5559.0 (3393.2–8733.2)	1580 (4.7)	4588 (16.7)	18,923 (78.7)
2013															
n (%)	39,676 (100.0)	5113 (31.4)	10,263 (21.7)	16,101 (34.7)	8199 (12.2)	22,986 (43.5)	10,042 (19.7)	6648 (36.8)	14,009 (34.5)	13,511 (34.9)	12,156 (30.6)	5851.9 (3615.1–9140.6)	1571 (3.8)	5230 (15.7)	23,652 (80.4)
2014															
n (%)	40,125 (100.0)	5267 (31.4)	10,204 (21.9)	16,418 (34.9)	8236 (11.8)	22,900 (43.7)	10,396 (19.2)	6829 (37.1)	14,636 (35.3)	13,554 (35.1)	11,935 (29.6)	6078.9 (3799.4–9531.5)	1378 (3.4)	5056 (15.0)	24,414 (81.6)
2015															
n (%)	39,863 (100.0)	5101 (32.4)	10,692 (22.3)	15,745 (33.9)	8325 (11.4)	23,016 (42.6)	10,204 (18.8)	6643 (38.6)	15,763 (38.4)	13,916 (35.0)	10,184 (26.6)	6333.3 (3971.9–9959.0)	1329 (3.2)	5153 (14.6)	25,706 (82.2)
2016															
n (%)	44,949 (100.0)	5306 (31.9)	13,579 (21.5)	17,249 (34.7)	8785 (11.9)	25,608 (44.0)	12,406 (18.7)	6905 (37.3)	17,716 (38.4)	15,472 (35.8)	11,731 (25.9)	6685.8 (4256.3–10,547.0)	1275 (2.9)	5221 (13.8)	29,289 (83.3)
2017															
n (%)	43,545 (100.0)	5434 (32.4)	12,237 (21.0)	17,047 (34.8)	8827 (11.7)	25,147 (43.5)	11,317 (18.8)	7081 (37.6)	17,676 (39.1)	14,479 (34.1)	11,390 (26.8)	6866.6 (4295.5–10,813.3)	1212 (2.9)	5246 (14.5)	28,126 (82.6)
2018															
n (%)	47,700 (100.0)	5458 (32.4)	12,842 (21.1)	19,791 (34.5)	9609 (12.0)	28,327 (43.9)	12,013 (18.8)	7360 (37.3)	19,856 (40.0)	15,106 (33.0)	12,738 (30.0)	7061.2 (4435.4–11,218.2)	1069 (2.2)	5680 (14.4)	30,713 (83.4)

% Proportions include the factor expansion and the complex survey design for each year of the ENAHO surveys

Q1-Q3: Quartile 1 - Quartile 3

The prevalence of access to safe drinking water during 2008 and 2018 across the country is presented in the Supplemental Material. In 2008, 46.7% of the households had access to safe drinking water, increasing to 51.9% in 2018 (difference in proportions between 2018 vs. 2008 5.2 percentage points, *p* < 0.001). For small cities, access to safe drinking water did not show changes between 2018 and 2008 (difference in proportions − 0.2 percentage points, *p* = 0.741); however, there was an increase in access to safe drinking water in medium (difference in proportions 3.3 percentage points, *p* < 0.001) and large cities (difference in proportions 12.8 percentage points, *p* < 0.001). The test for trend showed a change in access to safe drinking water from 2008 to 2018 for all city sizes (Table 2). All tests for trend estimations were confirmed by logistic regression analyses.

**Table 2:** Trends and differences in access to safe drinking water in Peruvian households, ENAHO 2008–2018

Year	General population (excluding rural areas)	City size		
		Small	Medium	Large
	% (95% CI)	% (95% CI)	% (95% CI)	% (95% CI)
2008	46.7 (45.90–47.47)	29.2 (28.32–30.17)	49.1 (47.70–50.42)	65.6 (64.08–67.07)
2009	43.0 (42.19–43.78)	22.5 (21.67–23.34)	42.9 (41.57–44.26)	66.0 (64.46–67.45)
2010	45.2 (44.42–46.01)	24.1 (23.29–24.98)	44.3 (42.91–45.59)	68.9 (67.46–70.34)
2011	48.4 (47.65–49.11)	27.8 (27.02–28.64)	44.9 (43.61–46.10)	74.3 (73.04–75.59)
2012	47.8 (47.05–48.53)	28.0 (27.22–28.89)	45.1 (43.86–46.37)	72.3 (70.98–73.61)
2013	50.8 (50.12–51.40)	28.1 (27.38–28.84)	49.2 (47.98–50.32)	78.4 (77.35–79.48)
2014	49.3 (48.68–49.98)	28.0 (27.32–28.81)	48.0 (46.84–49.21)	75.1 (73.97–76.22)
2015	46.8 (46.13–47.48)	23.0 (22.30–23.73)	45.6 (44.35–46.75)	73.7 (72.49–74.89)
2016	48.3 (47.64–48.93)	26.2 (25.51–26.89)	49.4 (48.18–50.52)	73.8 (72.62–75.02)
2017	49.1 (48.50–49.79)	27.4 (26.74–28.15)	48.9 (47.72–50.09)	74.4 (73.23–75.56)
2018	51.9 (51.23–52.48)	29.1 (28.36–29.74)	52.4 (51.25–53.55)	78.4 (77.31–79.49)
*P*-value, test for trends*	< 0.001	0.009	< 0.001	< 0.001
Difference 2018 vs. 2008				
Percentage points (95% CI)	5.2 (4.2; 6.2)	−0.2 (− 0.9; 1.3)	3.3 (1.6; 5.1)	12.8 (11.0; 14.7)

% include the factor expansion and the complex survey design for each year of the ENAHO surveys

The symbol (*) indicated the method to obtain the p-value

**Fig. 1:**
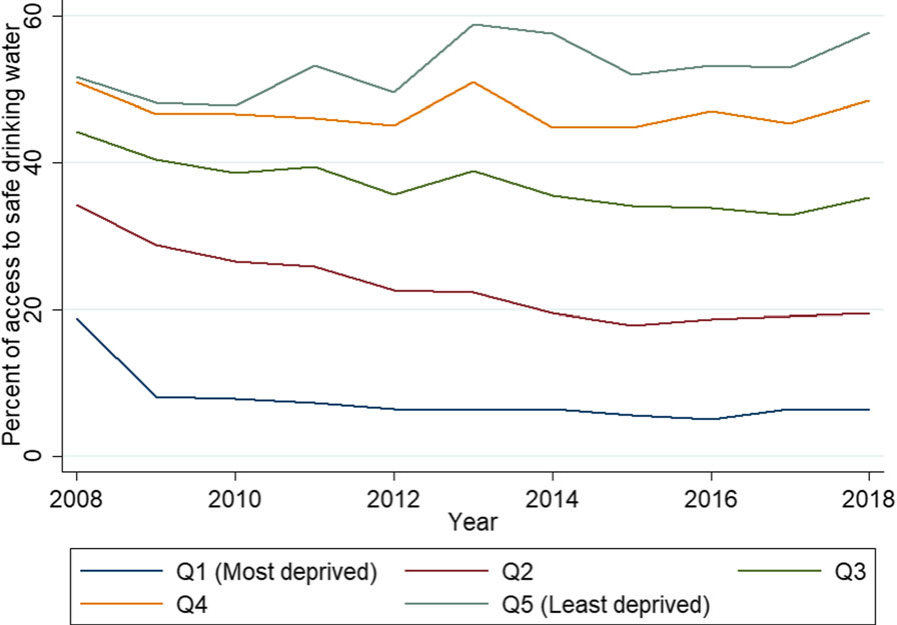
Access to safe drinking water in Peruvian households, excluding rural areas, according to quintile of household per capita expenditure, ENAHO 2008–2018

**Fig. 2:**
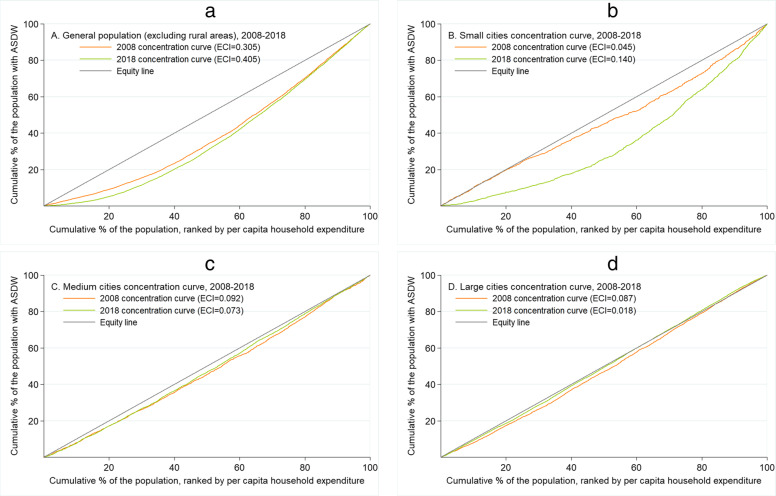
Concentration curves for access to safe drinking water in Peruvian households, ENAHO 2008–2018. **a** General population (excluding rural areas), 2008–2018. **b** Small cities concentration curve, 2008–2018. **c** Medium cities concentration curve, 2008–2018. **d** Large cities concentration curve, 2008–2018. Notes: ASDW, access to safe drinking water

According to the households’ per capita expenditure, from 2008 to 2018 the percent of access to safe drinking water shows an inequality between the most deprived (Q1 and Q2) and the least deprived (Q5) households (Fig. 1). Figure 2 shows CCs displaying inequalities in access to safe water in favor of the wealthiest population in the general population (panel A), and that the inequalities gap in year 2018 was wider in the small cities (panel B) than in medium (panel C) and large cities (panels D).

Regarding the ECIs, only positive values were obtained in the general population for the years included in the study, evidencing access to safe drinking water is concentrated in wealthiest households (Table 3). In the smallest cities the socioeconomic inequality appears to have increased (from ECI [2008] 0.045 to ECI [2018] 0.140, *p* < 0.001), while in larger cities socioeconomic inequality seems to be reduced (from ECI [2008] 0.087 to ECI [2018] 0.018, *p* = 0.036) (Table 3).

**Table 3:** Erreygers Concentration Index for access to drinking water in Peruvian households, ENAHO 2008–2018

Year	General population (excluding rural areas)		City size					
			401 to 10,000 households		10,001 to 100,000 households		≥ 101,001 households	
	**ECI**	**SE**	**ECI**	**SE**	**ECI**	**SE**	**ECI**	**SE**
2008	0.305	0.0145	0.045	0.0172	0.092	0.0220	0.087	0.0267
2009	0.373	0.0132	0.111	0.0112	0.075	0.0198	0.043	0.0283
2010	0.356	0.0123	0.085	0.0099	0.067	0.0203	0.026	0.0258
2011	0.383	0.0113	0.102	0.0091	0.073	0.0170	0.051	0.0232
2012	0.365	0.0114	0.103	0.0099	0.082	0.0177	0.023	0.0248
2013	0.436	0.0097	0.103	0.0076	0.107	0.0191	0.067	0.0204
2014	0.416	0.0100	0.096	0.0087	0.110	0.0183	0.107	0.0211
2015	0.382	0.0104	0.089	0.0079	0.106	0.0181	0.016	0.0216
2016	0.381	0.0096	0.113	0.0090	0.120	0.0180	−0.022	0.0209
2017	0.367	0.0097	0.110	0.0077	0.073	0.0178	−0.007	0.0215
2018	0.405	0.0092	0.140	0.0095	0.073	0.0185	0.018	0.0194
*P*-value*	< 0.001		< 0.001		0.517		0.036	

ECI Erreygers Concentration Index; SE standard error

* For differences between 2018 ECI vs. 2008 ECI

## Discussion

The study findings show a significant increase in the proportion of Peruvian households with access to safe drinking water during 2008–2018. Over the 11-year study period, the gap in access to safe water between large cities and smaller cities increased. Furthermore, regardless of the city size, the access to safe drinking water was concentrated in the wealthiest households, being more pronounced in the small cities. These results, including variation among and within cities, provide clear evidence for policymakers to monitor access to safe drinking water and to better target future resource allocation on increasing universal access, emphasizing on the unequal distribution of health resources as a challenge for national development, environmental justice and public health governance.

Over the 11-year study period, we found an increasing trend in the proportion of the Peruvian general population with access to safe drinking water. In addition, we also report a widening gap in the access to safe drinking water between the wealthiest and the poorest households over the same study period. These findings, in terms of the direction of the results, are similar to previous studies in Peru [17] and other low- and middle-income countries [18–23]. In addition, by using the ECI as a measure of inequality, we found that in small cities the ECI index was nearly eight times higher during 2018 compared to the ECI for big cities in the same year. The wider inequalities observed among small cities may be explained by a variety of factors including the heterogeneity of urbanization in terms of financial, human, and technological resources, as well as the availability of water sources. These characteristics, and the complex interactions between them, may affect the real capacity to meet the demand for water in cities. In other words, according to the theory of agglomeration economics [24] we can expect higher concentration of capacities in larger cities due to economies of scales and division of labor including more financial and human resources, which can lead to an urban advantage in comparison to smaller cities. This advantage in terms of capabilities, will in turn affect the capacity to reduce inequalities, including access to water. This is an important issue because it has been described that (the lack of) financial and human resources are major limitations that may jeopardize the development or deployment of drinking water strategies [25]. Another reason that can explain wider inequalities among small cities is the governance performance. Larger cities can facilitate coordination and enhance the power to organize actions for demanding, for example, access to safe drinking water, and can also promote the social skills and connections that collectively compose “civic capital”: the ability of citizens to improve the quality of their government [26]. Yet, further research is required to disentangle the relationship between governance performance on issues related to access to safe drinking water and city size. In addition to the differential effects observed between large and small cities, the gap in access to safe drinking water between wealthiest and poorest households may also be explained, among various other factors, by the unequal distribution of subsidies including the imperfect application of fees and tariffs. While the former favor those households with a pipe connection, thus leaving behind those households without a connection, the latter may yield to reduction in access to safe drinking water for those in the lowest quintiles of income [27].

Actions to improve universal access to safe drinking water should consider the city size. In larger cities, poor households are often confined to slums or areas without or limited municipal services, resulting in higher costs of water services [28]. Large urban areas with high rates of population growth due to natural population growth or rural-urban migration phenomena, have not planned growth in peripheral areas, where poor and low-income families tend to reside, creating challenges for the provision of water and sanitation that needs to be faced by national and local governmental strategies [29, 30]. Therefore, in large cities policies must deal with the increasing trend of population living in peripheral areas and face the challenge of maintaining the current safe water coverage [2, 22]. In the small cities, target interventions for those who need it the most, those in the lowest quintile index, can provide a greater impact, and thus create a value-for-money investment. Considering that small cities may need better infrastructure related to water and sanitation, [31] local-level investments in water and sanitation can be a suitable intervention to address the inequalities, and contribute to the prevention of communicable diseases, [32] increases life expectancy, and reduces infant and maternal mortality [33, 34], major problems that greatly affect poor and segregated populations.

Looking at the pattern of access to safe drinking water in the general population during the 11-year study period, the largest declines (≥ 2.5 percentage points) occurred during 2008–2009 and during 2014–2015. A possible explanation of the declines in access to safe drinking water during 2008–2009 and 2014–2015 may be related to that the governance and governability aspects of the Sanitation Service Providers (Empresa Prestadora de Saneamiento by its name in Spanish) fail to respond to water and sanitation needs of the urban population. In a study that used governance and governability index, of the 49 Sanitation Service Providers assessed in 2014, none of them were rated with good performance, while 18, 25 and 57% were rated with fair, bad and very bad performance [35]. The results of this study means that the Sanitation Service Provider have weaknesses that limit their capacity to meet the water and sanitation demand of urban population.

There are some limitations to consider when interpreting the present findings. The survey records the source of drinking water at households rather than individuals, thus limiting its ability to make causal inferences. Likewise, the outcome variable was measured only at one time per year, and we were unable to capture variability in water chlorination levels or changes in different stages of the year. Additionally, because ENAHO does not report information required to estimate per capita household expenditure adjusted by adult equivalent scale (age and sex of every household member), we cannot measure the household composition to take into account the economies of scale in consumption. Lastly, free residual chlorine measurements provided only a partial and indirect indicator of microbial contamination of piped water. Other exposures to inadequate chlorinated water is likely to be underestimated in the analysis. However, it is unclear how this limitation can affect the inequalities in access to safe drinking water across the population.

Despite the limitations, our study provides new insights into socioeconomic inequalities of the trend of access to safe drinking water across Peruvian households, according to the size of the city. Strengths of the study include the large sample drawn from nationally representative surveys over an 11-year period using standardized protocols and measurement tools. Therefore, our findings provide a comprehensive picture of the temporal trend of safe drinking water in Peru with generalizability to urban areas in the entire country and other countries with similar patterns of urban growth.

In conclusion, in Peru, inequalities in access to safe drinking water are still present, and access is greater for the wealthiest population and for those living in larger cities. Much of the findings of this research would be hidden if they were other spatial scales with regional or national averages, failing to show a reality on the local scale. Since the progress in access to safe drinking water during 2008–2018 is not equally distributed throughout the Peruvian population, priority to increase the access to safe drinking water should be given to socially disadvantaged groups and small cities. Promotion and support for effective implementation of policies and strategies to safe drinking water, including equity-oriented infrastructure development and resource allocation for most vulnerable settings, should be a priority.

## Supplementary Information

**Additional file 1: Figure S1.** Access to safe drinking water in Peru across departments, excluding rural areas, ENAHO 2008–2018.

## Data Availability

All data analyzed in this study is open access and can be obtained from the INEI’s website (http://iinei.inei.gob.pe/microdatos).

## Abbreviations

LAC: Latin American and the Caribbean
ENAHO: National Household Survey
INEI: National Institute of Statistics and Informatics
DPD1: Diethyl paraphenylene diamine
PEN: Peruvian Soles
ECI: Erreygers concentration index
CC: Concentration curves
