# Supplementary figures and images for: Inequalities in access to safe drinking water in Peruvian households according to city size: an analysis from 2008 to 2018
Source: Int J Equity Health. 2021 Jun 5;20:133. doi: 10.1186/s12939-021-01466-7 (PMC8178848; doi:10.1186/s12939-021-01466-7)

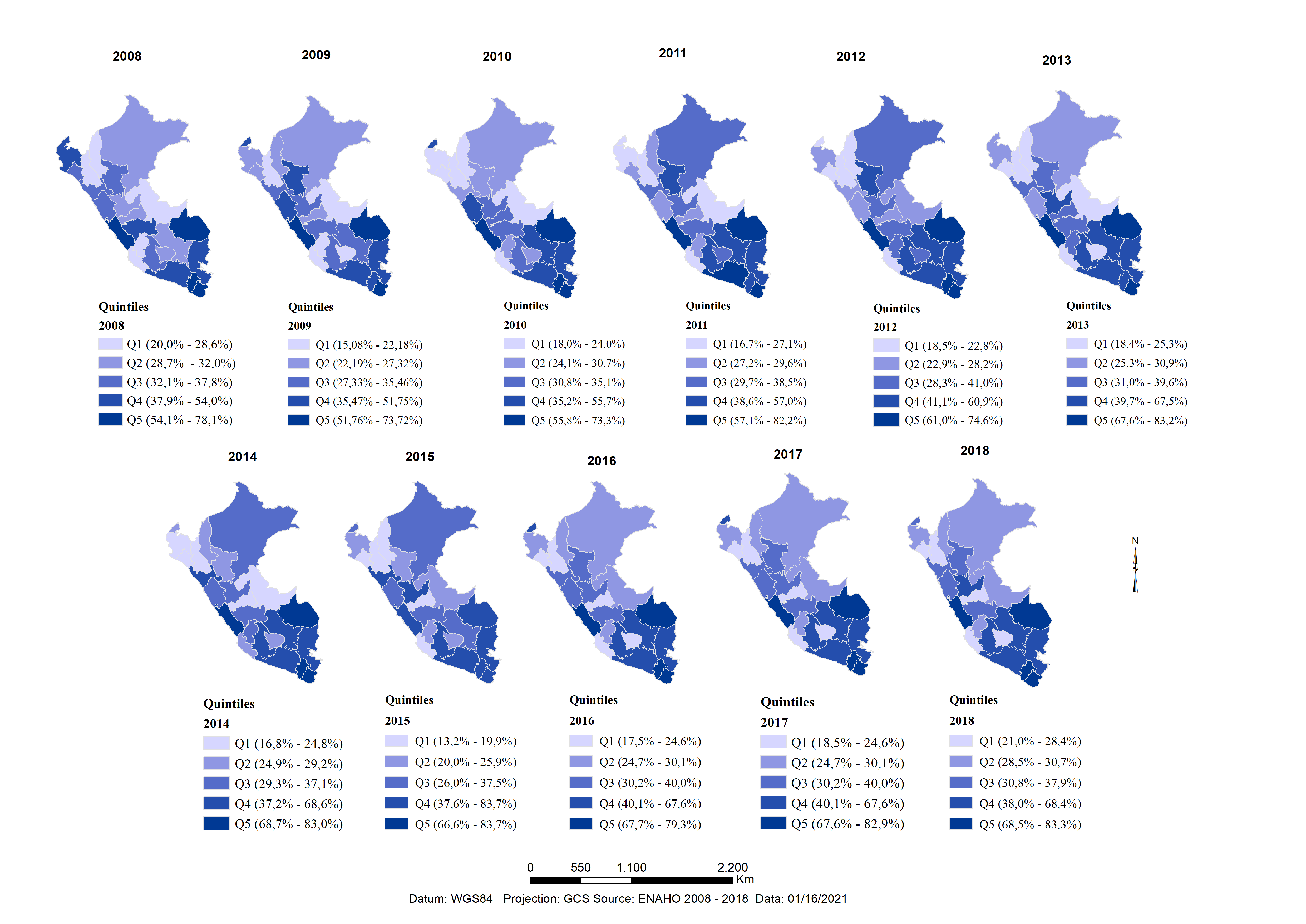

Supplement: Supplementary file 1 — Additional file 1: Figure S1. Access to safe drinking water in Peru across departments, excluding rural areas, ENAHO 2008–2018. [file 12939_2021_1466_MOESM1_ESM.zip › Supplementary_Map_Peru.tif]
